# Supplementary material for: Association of Hyperkalemia and Hypokalemia with Patient Characteristics and Clinical Outcomes in Japanese Hemodialysis (HD) Patients
Source: J Clin Med. 2023 Mar 8;12(6):2115. doi: 10.3390/jcm12062115 (PMC10058536; doi:10.3390/jcm12062115)
Supplement: Supplementary file 1 [file jcm-12-02115-s001.zip › jcm-2205328-supplementary.pdf]

SUPPORTING INFORMATION

Association of Hyperkalemia and Hypokalemia with Patient Characterstics and Clinical Outcomes in Japanese Hemodialysis (HD) Patients

Masao Iwagami[1,2], Yuka Kanemura[3], Naru Morita[3], Toshitaka Yajima[3*] Masafumi Fukagawa[4], Shuzo Kobayashi[5]

[1]Department of Health Services Research, Faculty of Medicine, University of Tsukuba

[2]Faculty of Epidemiology and Population Health, London School of Hygiene and Tropical Medicine

[3]Cardiovascular, Renal, and Metabolism, Medical Affairs, AstraZeneca K.K.

[4]Division of Nephrology, Endocrinology, and Metabolism, Tokai University School of Medicine

[5]Kidney Disease and Transplant Center, Shonan Kamakura General Hospital

Corresponding author

Toshitaka Yajima

E-mail: Toshitaka.Yajima@astrazeneca.com

Table of contents

	Page
**Table S1.** List of comorbidities	2
**Table S2.** Definitions of clinical outcomes	5
**Table S3.** Cumulative incidence of clinical outcomes in outpatient managed patients undergoing HD 3 times/week during baseline period	6

Table S1. List of comorbidities

**Comorbidity**	**Definition in ICD-10 code**
Heart Failure (HF)	I50, I110
Diabetes (DM)	E10-E14
Diabetic nephropathy	E102, E112, E122, E132, E142
Hypertension	I10-I15
Glomerular diseases	N05-N08
COPD	J44
Stroke	I60, I61, I63, I64
Myocardial Infarction (MI)	I21, I22
Atherosclerotic heart disease of native coronary artery	I25.1
Chronic ischaemic heart disease	I25.2, I25.8, I25.9
Other acute ischaemic heart diseases	I24.0
Unstable angina	I20.0
Angina pectoris, unspecified	I20.9
Peripheral vascular diseases	I70-I74, I77
Cerebrovascular diseases	I60-I69, G45
Dementia	F00-F03, F051, G30
Chronic pulmonary diseases	J40-J47, J60-J67, J684, J701, J703, J841, J920, J961, J982, J983
Connective tissue diseases	M05, M06, M08, M09, M30, M31, M32, M33, M34, M35, M36, D86
Ulcer diseases	K221, K25-K28
Mild liver diseases	B18, K700-K703, K709, K71, K73, K74, K760
Hemiplegia	G81, G82
Any Malignant tumour	C00-C75

Comorbidity	Definition in ICD-10 code
Leukaemia	C91-C95
Moderate to severe liver diseases	B150, B160, B162, B190, K704, K72, K766, I85
Metastatic solid tumour	C76-C80
HIV diseases	B21-B24
Atrial flutter	I483, I484
Atrial fibrillation	I480, I481, I482
Cardiomyopathy	I42
Heart valve diseases	I34, I35, I05, I06, I07, I08, I091, I099, I36-39
Alcoholism-related or other substance-abuse related disorders	F10-F19
Obesity	E66
Cardiac arrhythmia	I44, I45, I47-I49
Atrioventricular block	I440, I441, I442, I443
Bundle branch block	I444, I445, I446, I447, I451, I452, I453, I454
Cardiac arrest	I46
Paroxysmal supraventricular tachycardia, Atrial tachycardia, Re-entrant arrhythmia, WPW syndrome	I470, I471, I456
Ventricular tachycardia	I472
Torsade de pointes, Ventricular fibrillation	I490
Premature atrial contraction	I491
Premature ventricular contraction	I493
Sick sinus syndrome	I495
Oedema	R60
Sarcopenia	M625
Presence of cardiac pacemaker	Z950

Comorbidity	Definition in ICD-10 code
Polycystic kidney	Q612-Q613
Transplanted organ and tissue status	Z94

Abbreviation: ICD-10, International Classification of Diseases 10th revision

Table S2. Definitions of clinical outcomes

Outcome	Definition
All cause mortality	either in hospital death or in DPC hospital death
All cause Hospitalization	Record at least one hospitalization by any causes during the follow-up period.
Major adverse cardiovascular disease	Any of all cause mortality, myocardial infarction, or stroke<br>Myocardial infarction is defined as ICD-10 code I21 or I22.<br>Stroke is defined as ICD-10 code I61 or I63.
Cardiac arrest	ICD-10 code I46
Fatal arrhythmia	Ventricular tachycardia, Torsade de pointes, Ventricular fibrillation as ICD-10 code I472 or I490
Death related to arrhythmia	death within 3 days of fatal arrythmia

Abbreviations: HF, heart failure; ICD-10, International Classification of Diseases 10th revision

Table S3. Cumulative incidence of clinical outcomes in outpatient managed patients undergoing HD 3 times/week during baseline period

Clinical outcomes	Overall (n=2~~2,~~846)		Normo-K (n=1~~,~~624)		Hyper-K (n=909)				Hypo-K (n=313)			
	N	100 PY	N	100 PY	N	100PY	Crude HR (95% C.I)	Adjusted[a] HR (95% C.I)	N	100PY	Crude HR (95% C.I)	Adjusted[a] HR (95% C.I)
**All cause mortality**												
at 6 months	58	4.16	32	4.02	15	3.36			11	7.22		
at 1 years	166	6.10	89	5.73	49	5.62			28	9.48		
at 3 years	498	7.21	273	6.92	158	7.02			67	9.37		
at 5 years	727	7.60	392	7.21	240	7.55			95	9.99		
over entire follow-up	947	7.78	513	7.41	314	7.50	1.01 (0.87, 1.16)	0.92 (0.78, 1.09)	120	11.29	1.58 (1.30, 1.94)	1.47 (1.13, 1.92)
**MACE**												
at 6 months	125	9.08	60	7.61	37	8.39			28	18.90		
at 1 years	287	10.80	140	9.18	96	11.27			51	18.10		
at 3 years	727	11.10	392	10.41	236	11.11			99	15.07		
at 5 years	995	11.19	529	10.39	333	11.30			133	15.61		
over entire follow-up	1238	11.20	671	10.63	415	10.88	1.02 (0.91, 1.16)	0.97 (0.84, 1.12)	152	16.29	1.57 (1.31, 1.87)	1.48 (1.17, 1.86)
**Hospitalization**												
at 6 months	666	54.28	354	50.36	230	58.85			82	61.58		
at 1 years	1,068	49.12	582	46.33	360	52.63			126	53.89		
at 3 years	1,814	42.05	996	39.34	615	45.62			203	46.77		
at 5 years	2,076	40.52	1,151	38.22	704	43.70			221	44.12		
over entire follow-up	2,194	39.20	1,216	37.00	751	41.97	1.14	1.09	227	43.54	1.13	1.13

							(1.04, 1.24)	(0.98, 1.22)			(0.98, 1.31)	(0.94, 1.37)
**Cardiac arrest**												
at 6 months	7	0.50	4	0.50	2	0.45			1	0.66		
at 1 years	19	0.70	9	0.58	9	1.03			1	0.34		
at 3 years	56	0.81	27	0.68	23	1.02			6	0.84		
at 5 years	75	0.79	35	0.64	30	0.95			10	1.05		
over entire follow-up	105	0.86	54	0.78	41	0.98	1.24 (0.83, 1.86)	1.30 (0.80, 2.12)	10	0.94	1.31 (0.66, 2.60)	1.41 (0.58, 3.38)
**Fatal arrythmia**												
at 6 months	8	0.57	3	0.38	5	1.12			0	0.00		
at 1 years	17	0.63	8	0.52	9	1.04			0	0.00		
at 3 years	43	0.63	19	0.48	24	1.08			0	0.00		
at 5 years	60	0.63	32	0.59	26	0.83			2	0.21		
over entire follow-up	77	0.64	43	0.63	32	0.78	1.25 (0.79, 1.97)	1.44 (0.86, 2.39)	2	0.19	0.31 (0.08, 1.30)	0.28 (0.04, 2.16)
**Death related to arrythmia**												
at 6 months	1	0.07	0	0.00	1	0.22			0	0.00		
at 1 years	3	0.11	1	0.06	2	0.23			0	0.00		
at 3 years	12	0.17	3	0.08	9	0.40			0	0.00		
at 5 years	15	0.16	5	0.09	9	0.28			1	0.11		
over entire follow-up	19	0.16	7	0.10	11	0.26	2.65 (1.03, 6.84)	3.11 (1.03, 9.33)	1	0.09	0.98 (0.12, 8.07)	2.65 (0.31, 22.89)

To estimate the hazard ratio, the Normo-K group will be used as the reference level.
a Adjustments: age, gender, dialysis vintage, index year, body mass index (BMI), albumin, C-reactive protein (CRP), urea reduction ratio (URR), serum Ca, serum phos-phorus (centralized by subtracting mean), serum phosphorus squared (squared after centralized), hemoglobin (HGB), HD treatment time, Charlson Comorbidity Index (CCI), and history of comorbidities: heart failure, diabetes, cardiovascular comorbidities, myocardial Infarction, other acute ischemic heart diseases, atherosclerotic heart disease of native coronary artery, chronic ischemic heart disease, stroke, unstable angina, angina pectoris unspecified, hear failure, and diabetes.
